# The Devil is in the Framework. Comment on Mizrahi vs. all Debate on the Strength of Arguments from an Expert Opinion

**DOI:** 10.1007/s11406-022-00490-3

**Published:** 2022-04-20

**Authors:** Szymon Makuła

**Affiliations:** Faculty of Humanities, University of Silesia, Katowice, Poland

**Keywords:** Argument from expert opinion, Argument strength, Examination dialogue

## Abstract

In one of his papers, Moti Mizrahi argues that arguments from an expert opinion are weak arguments. His thesis may seem controversial due to the consensus on this topic in the field of informal logic. I argue that its controversy is framework-dependent, and if translated into a different framework, it appears to be a correct, however trivial, claim. I will use a framework based on Douglas Walton’s argumentation scheme theory and his conception of examination dialogue to demonstrate that it is so. It appears that Mizrahi’s idiosyncratic framework provides an excessively restrictive conception of an argument from expert opinion than Walton’s scheme does. There is no quarrel between both frameworks, as they yield analogous, almost identical, outcomes of argument evaluation. The actual and crucial disagreement is on the topic of argument classification. Mizrahi’s conception of arguments from an expert opinion imposes exact conditions that such argument must fulfil: an expert’s opinion *o* truth-value must be unknown; *o* must be unsupported by any evidence; an expert’s peers neither accept *o* nor reject it. These exclude, by definition, every possible strong, in Walton’s terms, variant of such an argument. Therefore, if rephrased with the notions of the examination dialogue framework, Mizrahi’s thesis sounds as follows: weak arguments from expert opinion are weak arguments.

## Introduction

There is an overwhelming consensus on the strength of arguments from expert opinion (AEO hereafter) among logicians, argumentation theorists, and authors of critical thinking textbooks (Copi et al. 2018; Govier 2013; Kahane 1998). AEOs are regularly considered plausible arguments; of course, plausibility comes under certain conditions. The degree to which these conditions are met is why AEO differs in strength. As a result, AEO yields a broad class of arguments, from irrefutably fallacious kinds such as *argumentum ad verecundiam* to those that support a conclusion adequately, which makes them acceptable.

According to Walton, Reed, and Macagno (2008, pp. 91), one of the most basic schemes of AEO is as follows^1^:

P1: Source E is an expert in subject domain S containing proposition A.

P2: E asserts that proposition A is true (false).

C: A is true (false).

The above scheme is a formula for any possible AEO occurring in natural language argumentation. It incorporates two premises (P1 and P2) and a conclusion (C). P1 claims that person E is an expert in a domain that is definite and crucial for the subject under discussion; P2 accredits proposition A to the specified in P1 expert E. If any argument is reducible to the above set of P1, P2, and C, then it should be included in the AEO class and evaluated as such. AEOs are so-called presumptive arguments, meaning their acceptance is provisional and subject to subsequent rejection (Walton 1997). Critical questions are a means of evaluating AEO, and AEO is linked to an appropriate list of such questions as any other argumentation scheme^2^. The final grade assigned to a given AEO varies from weak to strong, depending on the number and quality of responses obtained to the relevant critical questions (Walton 2014). It follows that the overall strength of AEO is a continuum that falls between these two poles.

Despite unanimity on the strength of AEO, Moti Mizrahi (2013) wrote a paper whose central thesis was that AEOs are weak arguments. His article started a long debate between the author and a small army of scholars. In contrast to most of Mizrahi’s opponents, I will argue that his thesis is correct; however, it is trivial and uncontroversial. The whole discussion induced by Mizrahi’s papers is based on a profound oversight regarding an argumentation framework he used. In order to demonstrate that this is so, I intend to transcribe Mizrahi’s framework into a framework based on Douglas Walton’s (2006) concept of examination dialogue.

## Debate on Mizrahi’s Paper

As mentioned above, Moti Mizrahi (2013) questioned the received view on AEO strength by arguing that AEOs are weak arguments. He means that the premises of AEO provide insufficient support for the conclusion (Mizrahi 2013). Therefore, despite the apparent truth of both premises, the conclusion is not acceptable. Mizrahi’s (2013, p. 58) argument runs as follows:

1. Arguments from expert opinion are weak arguments unless the fact that expert E says that p makes it significantly more likely that p is true.
2. [As empirical evidence on expertise shows] the fact that E says that p does not make it significantly more likely that p is true.
3. Therefore, arguments from expert opinion are weak arguments.

For Mizrahi’s line of argumentation, premise (2) is the most crucial. He supports it with many studies on expert credibility, enriched further with additional items in his subsequent article (Mizrahi 2013; Mizrahi 2018). As Mizrahi (2013, p. 76) affirms, “research on expertise shows that expert opinions are only slightly more accurate than chance,” which makes premises (1) and (2) acceptable. Therefore, he concludes that AEOs are weak arguments.

This article was met with a wave of criticism and polemics. Markus Seidel (2014) provided five arguments against Mizrahi’s thesis. Douglas Walton (2014) asserts that Mizrahi analyzes an oversimplified single-premise AEO scheme, completely ignoring the additional premises provided by answers to critical questions. Martin Hinton (2015) takes on both Mizrahi’s paper and Seidel’s critique, as he examines the confusion caused by the ambiguity of such notions as “expert” and “opinion;” in conclusion, he argues that Mizrahi’s argument should be re-worded in a manner where instead of the notion of “expert opinion,” the phrase “expert prediction” is used. Mizrahi replies to Seidel’s criticism by pointing to a significant misunderstanding, as his opponent “fails to distinguish between administrative (or practical) authority and cognitive (or epistemic) authority, between epistemic trust and professional trust, and between judgments and procedures” (Mizrahi 2016, p. 250). Nevertheless, misinterpreting or not, Seidel fails, in Mizrahi’s opinion, to demonstrate that AEOs are strong arguments (Mizrahi 2016). Meanwhile, on a side avenue of this debate, Seidel (2016) confronts Hinton’s criticism, arguing, among other things, that his accusation of Seidel’s claims being self-contradictory is based on misinterpretation, and therefore ill-targeted. David Botting (2018) argues that Mizrahi’s point fails to distinguish between two different types of arguments from positions to know, more specifically inductive and non-inductive ones. As he concludes, these can be, in some cases, strong arguments. Mizrahi (2019) replies to Botting’s objections, which due to incommensurable terms such as “weak argument,” missed the intended target, namely premise (1). Last but not least, a paper by Yanlin Liao (2021) argues that both of Mizrahi’s arguments fail because of inconsistency, irrelevance, and insufficiency^3^.

As much as this debate is engaging and could be an exciting research topic on its own, I intend to focus mainly on Mizrahi’s framework. Any fruitful discussion must be grounded on the established and approved conceptual framework, which provides unambiguous terms, concepts, and notions. Otherwise, as Mizrahi noted, “we would be talking past each other” (Mizrahi 2019, pp. 108), words are exchanged but not interchanged. That usually occurs when there are divergent frameworks unknowingly in use. I intend to argue that this is somewhat the case in the Mizrahi vs. all discussion. Therefore, this goal of this article is first and foremost to reconstruct Mizrahi’s idiosyncratic framework.

## Mizrahi’s Framework

Mizrahi’s (2013) point is straightforward at first sight: AEOs are weak arguments, period. However, to raise any objection or proper criticism, one should use terms with the same meaning as Mizrahi’s. Therefore, the meaning of such terms as AEO and “weak arguments” must be unambiguous. As AEO scheme Mizrahi (2013, pp. 61) introduced the following inference:

(P) Expert *E* says that *p*.

(C) Therefore, *p*.

Mizrahi (2013; 2016) made a few essential caveats. Foremost, Mizrahi understands opinions as predictions, judgments, decisions, or diagnoses. These are distinguished from expert knowledge, empirical evidence gathered by experts, or even artificial procedures governing decision-making (Mizrahi 2013; 2016). By definition, proposition *p* is a mere opinion; thus, its truth value is unknown^4^ (Mizrahi 2013, p. 58). Put simply, besides that expert E said so, there is no other reason to accept *p* (Mizrahi 2013, p. 61). Furthermore, Mizrahi’s AEO scheme should be strictly demarcated from different kinds of appeals to authority. Among the excluded arguments are those in which the premise reports what the majority of experts accept (Mizrahi 2013, pp. 61, 69); arguments in which the premise states that what expert says is based on evidence (Mizrahi 2013, 69); arguments from expert knowledge in which the premise posits that an expert knows that *p* (Mizrahi 2016, p. 241). Mizrahi (2013, p. 69) distinguishes such arguments with separate names, such as appeals to an agreement among experts or appeals to evidence.

Therefore, in Mizrahi’s framework, AEO pictures an argument in which conclusion *p* is backed up only with nothing more than an opinion of a single expert. That means *E* does not even know that *p*; she simply believes it. There is also not the slightest evidence to support *p*; there is also no agreement among peer experts on *p*. If there is, then such an argument is no longer AEO, because it appeals not only to an expert opinion but to evidence or consensus (Mizrahi 2013, 2016, 2018). I assume that the lack of a consensus among peer experts on whether to accept or reject *p* is due to its truth-value being unknown and the absence of supporting evidence. There are possible scenarios in which experts’ opinions are divided, as some experts accept *p*; few reject it, others are uncertain. It is even probable that no single expert besides the one quoted has any opinion on *p*. What matters is that there is no agreement among experts on *p* truth-value. In that case, proposition *p* is the sole belief of an individual expert unsupported by nothing but her hunches, guesses, intuitions, feelings, or instincts. Accordingly, AEO should be synonymous with arguments from an expert hunch, guess, intuition, feeling, or instinct.

As the meaning of AEO in Mizrahi’s framework is now settled, it is time for the “weak argument” notion. Contrary to his idiosyncratic AEO scheme, he contributes to the standard conception of a weak non-deductive argumentation, as “an argument in which the premises, even if true, provide weak support—or no support at all—for the conclusion” (Mizrahi 2013, p. 61). In other words, an argument is weak when its premises do not make its conclusion more likely to be true. As an example of such, Mizrahi (2019, p. 108) presents the following argument:

(P1) Few Americans are billionaires.

(P2) Jeff Bezos is an American.

(C) Therefore, Jeff Bezos is a billionaire.

Premises (P1) and (P2) are both true propositions. Yet, they provide insufficient support to the conclusion because the likelihood that any given American is a billionaire is low since only a few Americans are billionaires (Mizrahi 2019, p. 108). Strictly speaking, it is altogether more likely that a random American is not a billionaire; hence conclusion (C), although true, is more likely to be false. As an example of a strong argument, Mizrahi (2019, p. 109) presents the subsequent case:

(P1) Most Americans live in poverty.

(P2) Jeff Bezos is an American.

(C) Therefore, Jeff Bezos lives in poverty.

The above argument is stronger than the previous one; it is more likely for the conclusion to be true than false, given the truth of the premises. Since most Americans live in poverty, a random American is more likely to be poor than a billionaire. Mizrahi’s strength of an argument framework is all about inductive support, which has to do with “the probability of a conclusion being true given the truth of the premises” (Mizrahi 2019, p. 109). The first conclusion is true, and the second one is false; however, if the strength of an argument is considered, the truth-value of a conclusion is not as crucial as its conditional probability is.

To summarize the above, reconstruction of Mizrahi’s conceptual framework facilitates interpretation, making his thesis on the weak power of AEO more comprehensible. What he argues is, according to his framework, that: the whole AEO class is a set of arguments in which (a) expert opinion truth-value is unknown, as it is (b) unsupported by any evidence, and his (c) peers neither accept it nor reject it. Therefore, such arguments provide weak inductive support for a conclusion, which means that the probability of a conclusion being true given the truth of the premises is low.

That is because every single AEO satisfies conditions (a), (b), and (c), by definition. If a given argument references any empirical evidence, then it is a different type of argument, not an AEO; the same is for quoting consensus among experts. Mizrahi (2013; 2018) cited rich empirical evidence, indicating that expert opinions are scarcely better than non-expert guesses, their judgments are biased, and so on; therefore, AEO are weak arguments.

## Examination Dialogue Framework

Examination dialogue is a concept presented initially by Douglas Walton and Erik Krabbe (1995) and subsequently developed by its author and other scholars (Dunne et al. 2005; Walton 2006). Its primary purpose is to describe a specific type of dialogue in which one agent questions another and tries to find out what her interlocutor knows about something (Walton 2006). For example, dialogues of this type are characteristic of legal argumentation in trials when an attorney examines the testimony of an eye-witness or an expert opinion (Walton 2006).

One can ask how this idea is relevant to AEO’s strength? Arguments that fit the AEO scheme (both Walton’s and Mizrahi’s) are experienced wholly in so-called real-life dialogues expressed in natural languages. If these are a part of the equation, it is worth noting that they come with much baggage full of ambiguities, inconsistencies, vague and ill-defined terms, or equivocations. Such logical carelessness of natural languages is echoed considerably in real-life argumentation. Therefore, arguments in natural languages call for tools adequately adjusted to the medium’s distinguishable traits. That is why the examination dialogue concept, which has been developed along with argumentation scheme theory, prominent among scholars in the field of informal logic, is quite useful as a base for a conceptual framework of AEO and its strength.

As Walton (2006, pp. 746) puts it, examination dialogues have two goals: “the extraction of information and the testing of the reliability of this information.” The term “examination dialogue” can be misleading in two separate ways, demanding clarification. First, Walton has narrowed it down to examining only appeals to expert opinions, and it is used in this restricted meaning in this article. Second, it implies at least two participants. However, in most cases of AEO usage, there are three agents involved, at least indirectly (Walton 2006, pp. 755). There is no AEO without an expert holding a given opinion, and there is no AEO without a recipient of such an appeal. Experts, typically, are not physically present during the dialogue; in such cases, they are quoted by other parties supporting a similar point of view, which adds a third agent. However, a single person can indulge in an examination dialogue alone, especially in cases like an AEO in a newspaper article or a video. Hence, examination dialogue needs at least one information seeker and one expert opinion to be examined.

Examination dialogue invariably starts when there is a conflict of opinion, and one side argues by appealing to a view held by a given expert (Walton 2006, p. 755). Meanwhile, critical discussion emerges, both arguments and counterarguments are presented, critical questions are asked and answered. As mentioned above, the two goals of examination dialogue are information extraction and testing its reliability. These are nearly impossible to achieve without the correct recognition of arguments, although, in this case, we are, by definition, limited to the AEO. To correctly identify a given argument as an AEO, one must be familiar with its scheme. There are different ways of formulating such arguments because their schemes come with many alterations due to their perpetual development (Walton 1989, 1995, 1997, 2006; Walton et al. 2008). I shall rely on the one already quoted:

P1: Source E is an expert in subject domain S containing proposition A.

P2: E asserts that proposition A is true (false).

C: A is true (false).

The actual number of premises included in the AEO scheme may differ depending on the purpose; for example, it is suitable to use even Mizrahi’s one-premise scheme for educational purposes (Walton 2014). The argumentation scheme provides a clue that facilitates recognizing the type of a given argument and, consequently, selecting a matching list of critical questions. Hence, identifying the latter is much more significant than the actual list of premises included in the scheme. That is because critical questions are tools designed for examining and exploring additional information, if there is any. The correct answer to a given question usually appears as a hidden or extra premise of a given argument (Walton 2014; Walton et al. 2008). Such an answer serves as a means to evaluate it. That is why critical questions are much more crucial for argument analysis than the exact number of premises in the scheme. The strength of an AEO scales with the number of appropriately answered questions, as it tends to “get stronger and stronger” (Walton 2014, pp. 149) with subsequent accurate answers. A definitive set of critical questions matching the above AEO scheme is the following (Walton 1997, pp. 223):

Expertise Question: How credible is E as an expert source?

Field Question: Is E an expert in the field F that A is in?

Opinion Question: What did E assert that implies A?

Trustworthiness Question: Is E personally reliable as a source?

Consistency Question: Is A consistent with what other experts assert?

Backup Evidence Question: Is E’s assertion based on evidence?

As Walton (2006, pp. 750) puts it, the above six questions “are the gateway through which the [examination] dialogue is filtered.” Consider the following example of dialogue between person A and B. A said that:

1. “E is an MD, and she claims that COVID-19 vaccines are not safe, so they are not safe”.

B recognizes that (1) is an AEO, as it could be translated into an argumentation scheme:

1. P1: E is a medical expert.
  P2: E asserts that COVID-19 vaccines are not safe.
  C: COVID-19 vaccines are not safe.

After that, she can ask critical questions from the above list. That is when examination dialogue is launched. As B gets further appropriate answers during this dialogue, it results in a more sophisticated version of (AEO1), enriched with information B collected. Argument (1) is, as it turns out, a veil hiding compound inference, the strength of which depends on the number of hidden premises revealed by the critical questions answered. (AEO1) might transform into a more complex structure, which includes expertise premise (EP), field premise (FP), opinion premise (OP), trustworthiness premise (TP), consistency premise (CP), and backup evidence premise (BEP). Therefore, the outcome of the examination dialogue analysis of (1) is somewhere on the spectrum between the following two different inferences:

(AEO2) P1: E is a medical expert.
  P2: E asserts that COVID-19 vaccines are not safe.
  EP: E is not a credible source.
  FP: E is not a vaccinologist; she is a pediatrician.
  OP: E asserted that she is not sure if vaccines are safe for children.
  TP: E is not personally reliable as a source.
  CP: Opinion “COVID-19 vaccines are not safe” is inconsistent with what other experts assert.
  BEP: E’s assertion is not based on evidence.
  C: COVID-19 vaccines are not safe.

(AEO3) P1: E is a medical expert.
  P2: E asserts that COVID-19 vaccines are not safe.
  EP: E is a credible source.
  FP: E is a vaccinologist.
  OP: E asserted exactly that COVID-19 vaccines are not safe.
  TP: E is personally reliable as a source.
  CP: Opinion “COVID-19 vaccines are not safe” is consistent with what other experts assert.
  BEP: E’s assertion is based on evidence.
  C: COVID-19 vaccines are not safe.

(AEO2) and (AEO3) are, to say, two opposing poles of the potential (AEO1) strength, as (AEO2) is the weakest, even fallacious example of possible (AEO1) ’s variation, then (AEO3) is the strongest one there is. Suppose the examination dialogue outcome puts (AEO1) close to (AEO2), as it enriches (AEO1) with EP, FP, OP, TP, CP, and BEP like those included in (AEO2). In that case, it is not a good argument because the likelihood that an opinion of a noncredible, unreliable, and misquoted pediatrician is also unsupported by evidence and is inconsistent with the position of a vaccinologist’s community is true is close to 0. Suppose the examination dialogue outcome puts (AEO1) close to (AEO3) in a matter like the one above. In that case, it means that it is a good argument because the likelihood that an opinion of a credible and reliable vaccinologist, supported by evidence, consistent with a position of other vaccinologists, is true is close to 1^5^.

Summarizing the examination dialogue framework differs from Mizrahi’s in crucial aspects. Foremost, the AEO scheme provides a means for recognizing and matching an appropriate set of critical questions to a given argument. Any given AEO is evaluated in a context-dependent manner during an examination dialogue (Walton 2006, pp. 752), and the outcome of such dialogue revolves around its current stage. Argumentation, in this perspective, tends to be an open structure in which gaps are filled with content amid the examination dialogue. Some questions may remain unanswered due to the lack of dialogue participants’ knowledge suitable for the given critical question. Such gaps delay the argument’s analysis. Therefore, adequate evaluation is possible only if all critical questions are answered, which is often an unattainable case at the exact moment in which examination dialogue takes place. Such evaluation, in some sense, is never finished, as it is possible to obtain, at any time, new information which refutes an already accepted conclusion. Hence, rating any argument as weak or strong cannot be asserted in a context-free manner (Walton 2006, pp. 752). It is always based on specific answers to critical questions, collected in a unique examination dialogue.

Most importantly, these two frameworks differ on the AEO strength. As Mizrahi placed AEO among weak or even fallacious arguments, examination dialogue finds them on a vast spectrum; some are weak, some are strong, and some are somewhere in between. Hence, the following question needs to be answered: How does Mizrahi’s thesis on AEO strength translate into an examination dialogue framework?

## Mizrahi’s Thesis in Dialogue Examination Framework

Both frameworks, Mizrahi’s (AEO_MF_ hereafter) and dialogue examination (AEO_DEF_ hereafter), agree on the basic structure of the AEO scheme. What differentiates them is that AEO_DEF_ acknowledges multiple-premise variations of the AEO scheme, and AEO_MF_ shortens itself to only a one-premise version. It follows that every argument matching the following scheme is both AEO_MF_ and AEO_DEF_:

(P) Expert *E* says that *p*.
(C) Therefore, *p*.

However, some arguments, such as (AEO3), are included in AEO_DEF_ but excluded by AEO_MF_ as non-AEO. This claim is supported by the fact that the AEO_MF_ scheme is restricted not only to one-premise arguments but also to those that satisfy the following conditions, mentioned in Sect. 3:

- expert opinion *o* truth-value is unknown;
- expert opinion *o* is unsupported by any evidence;
- expert peers neither accept *o* nor reject it;

It must be highlighted that if any argument does not fulfill at least one of the above requirements, then it is not an AEO in AEO_MF_. However, such an argument still counts as an AEO in AEO_DEF_. Assuming the reconstruction of Mizrahi’s framework in Sect. 3 is correct, then, without doubt, every AEO in AEO_MF_ falls in the subcategory of AEO class in AEO_DEF_. This relation is not valid the other way around, as there are such AEO in AEO_DEF_, which are contained as different (non-AEO) arguments in AEO_MF_. Hence Mizrahi’s thesis on AEO being weak affects only a subset of such arguments in AEO_DEF_. This issue is mainly conceptual and framework-dependent. Accordingly, Mizrahi’s view should be interpreted in such a manner. If Mizrahi’s thesis is restricted to an idiosyncratic type of AEO, described in his framework, then it should be rephrased as:

(MF1) “All AEO that match AEO_MF_ conditions are weak arguments.”

According to the fact that arguments matching the AEO_MF_ scheme are a subset of an AEO_DEF_ class, then (MF1) is interchangeable with:

(MF2) “Some AEO by AEO_DEF_ standards are weak arguments.”

or more precisely:

(MF3) “Every AEO by AEO_DEF_ standards that simultaneously counts as an AEO by AEO_MF_ conditions is a weak argument.”

That is, of course, from the AEO_MF_ point of view. How does AEO_DEF_ evaluate the strength of its AEO_MF_ subset? According to AEO_DEF_, to evaluate AEO is to engage in an examination dialogue, during which one seeks any possible additional premises such as EP, FP, OP, TP, CP, and BEP. The actual strength of a given argument depends on the gathered information, that is, the content of the additional premises mentioned. Such evaluation results place the given argument on a scale between two polarized arguments, for example (AEO2), which marks the weakest possible outcome, and (AEO3), a model for the strongest one. As AEO_MF_ provides a particular type of AEO, it is possible to estimate the strength of the whole class relying only on its characteristics.

As the premise of an AEO_MF_ scheme claims, some expert has said that *p*, therefore, like any other expert she is credible or not, she is an expert in an appropriate field or not, she is misquoted or not, she is reliable or not, her opinion consistent with what other experts assert or not, her opinion is based on evidence or not. All these issues are one by one connected to the AEO’s critical questions. However, some of them are fixed by the AEO_MF_ characteristic of AEO. Both the consistency question and the backup evidence question are immediately out of the picture because if a given argument complies with AEO_MF_, it must fulfill conditions (a), (b), and (c). Condition (b) undermines backup evidence, and (c) undercuts consistency. Therefore, every AEO by AEO_MF_ standards subjected to an examination dialogue will end with CP such as “*p* is not consistent with what other experts assert” and BEP “*p* is not based on evidence.” However, it is open to interpretation whether the AEO_MF_ scheme allows quoting an opinion of an expert in an inappropriate field. I assume that such an argument falls in an appeal to the non-expert category. Therefore, FP is another fixed premise. As Mizrahi (2013, 2018) argues, all experts are not credible (at least not significantly more credible than non-experts) as sources of opinion, given they do not provide any empirical evidence and are not referring to decision procedures or quote consensus among peers. This claim provides the EP “E is not a credible source” and leaves the remaining two critical questions on opinion and trustworthiness.

Consequently, these four fixed issues burden every argument with three unacceptable premises: EP, CP, and BEP (FP is an acceptable one). Accordingly, if any argument matches the AEO_MF_ concept, it cannot be, by definition, a strong argument by AEO_DEF_ standards. Assuming that the remaining two critical questions are answered in favor of a given argument, it assembles the following inference^6^:

(AEO4) P1: Expert *E* says that *p*.
  *EP: E is not a credible source.
  *FP: E is an expert in the field F that *p* is in.
  OP: E asserted *p*.
  TP: E is personally reliable as a source.
  *CP: *p* is inconsistent with what other experts assert.
  *BEP: E’s assertion is not based on evidence.
  C: Therefore, *p*.

(AEO4) is a scheme for arguments appealing to the opinion, unsupported by evidence and not shared by other experts in the field, of a personally reliable, uncredible (e.g., biased) expert. Only three critical questions are answered correctly, providing the following acceptable premises: the expert is neither misquoted nor misinterpreted, personally reliable, and his expertise is in the appropriate field. Such inferences yield weak support for a conclusion, as it is not significantly more likely that the conclusion will be true given the premises being true. Any other possible variant^7^ of AEO_MF_ will be weaker than (AEO4), to the point being fallacious, as an equivalent of an (AEO2). To use Mizrahi’s (2013, p. 65) analogy, arguments based on (AEO4) scheme are like a “thermometer that gets the temperature right 55% of the time”; it is better than a guess. Still, it is not significantly superior for it to be relied on. It follows that any AEO that matches the AEO_MF_ scheme is between fallacious (no support for the conclusion) and weak (weak support) according to AEO_DEF_. If any AEO that is AEO_MF_ is not stronger than such a weak argument as (AEO4), then the (MF3) thesis is acceptable from the AEO_DEF_ point of view. Therefore, both frameworks will evaluate such arguments as weak, meaning arguments in which the premises being true does not make the conclusion significantly more likely to be true than false.

How about arguments that are strong AEO according to AEO_DEF_ standards but are excluded as AEO in AEO_MF_? Consider the following scheme:

(AEO5) P1: Expert *E* says that *p*.
  EP: E is a credible source.
  FP: E is an expert in the field F that *p* is in.
  OP: E asserted *p*.
  TP: E is personally reliable as a source.
  CP: *p* is consistent with what other experts assert.
  BEP: E’s assertion is based on evidence.
  C: Therefore, *p*.

(AEO5) is a model for the strongest possible AEO according to the AEO_DEF_ standards. Such inference provides strong support for the conclusion, as it is more likely that *p* is true than false, given that all premises are true. However, Mizrahi’s restrictive approach categorizes the above scheme as a different type of argument since it contains appeals to evidence and agreement among peer experts. Such arguments do not appeal to a mere opinion because the opinion is not the sole reason for accepting a conclusion (Mizrahi 2013, p. 71). Hence any argument with a CP or BEP is, according to AEO_MF_, a different scheme than AEO. I assume that (AEO5) should be treated as a combination of two different arguments (e.g., appeal to evidence and agreement among experts). Consider two variations of (AEO5), one in which there is no acceptable CP and the second without BEP. Both are strong arguments but weaker than (AEO5), at least according to AEO_DEF_ standards. Mizrahi seems to implicitly count such arguments as (AEO5) and its above modifications as strong because their strength is contrasted in his paper with the strength of AEO (Mizrahi 2013, p. 69). It appears that both frameworks agree on this issue.

The discussed frameworks assume slightly different and conflicting concepts of AEO. However, the AEO notions employed in both frameworks are mutually interpretable. Mizrahi’s concept is narrower than the one based on the dialogue examination framework. It follows that every AEO matching the AEO_MF_ scheme counts as an AEO in AEO_DEF_, and the AEO_MF_ scheme-based set of arguments is the shared part of both frameworks. These comply with all arguments matching the AEO_MF_ scheme being weak. Both frameworks recognize that every strong AEO, according to the AEO_DEF_ standard, is a strong argument, even if it is not an AEO under AEO_MF_ terms.

## Hinton and Seidel on Mizrahi’s Idiosyncratic Framework

The claim that debate on Mizrahi’s thesis is caused by an idiosyncratic framework has been proposed earlier, and it is partially discussed in Hinton’s (2015) and Seidel’s (2016) articles. My point may sound similar to Hinton’s approach; however, there are slight but crucial differences.

Hinton’s paper tackles the debate between Mizrahi’s (2013) first article and the response given by Seidel (2014). He aims to demonstrate that confusion between mentioned authors arose due to the distinct and implicitly restrained characterizations of the “expert opinion” notion (Hinton 2015, p. 540). Hinton and Seidel both agree that Mizrahi’s paper implied that AEO is reserved to a particular and narrow-ranged meaning of the term “expert opinion,” which in this case is equivalent to an expert prediction (Hinton 2015; Seidel 2014; Seidel 2016). Undoubtedly, Mizrahi uses “opinion” in a very narrow meaning, and this fact is the foundation upon which the idiosyncratic scheme of the AEO stands (Hinton 2015). As Hinton claims, there is a misunderstanding between Mizrahi and Seidel; however, more importantly, there is a prior misunderstanding, namely Mizrahi’s misconception of the AEO scheme.

Both Hinton and Seidel overlooked that Mizrahi’s framework yield analogous, almost identical, to Walton’s scheme outcomes of argument evaluation. If Mizrahi’s idiosyncratic framework is adjusted to a commonly used one, there is no need to quarrel, as his thesis is compatible with the established consensus on AEO strength. It appears that AEO_MF_ is just a specific, and well-defined for that matter, subset of AEO_DEF_, which is considered as a scheme for weak arguments by both frameworks. Therefore, it is meaningless to ponder such issues as “what is expertise,” as the whole debate Mizrahi vs. all is verbal. Moreover, Mizrahi finds strong AEO in AEO_DEF_ as strong arguments, supporting my claim that the entire dispute suddenly becomes nothing more than logomachy, even further.

## Conclusions

Discussed frameworks, both AEO_DEF_ and AEO_MF_, bring two significantly different classes of AEO to the table. Depending on which framework we choose, we employ a vast AEO class (AEO_DEF_) or utilize a narrow one (AEO_MF_). Notably, the former includes the latter, which means that if any argument counts as AEO in AEO_MF,_ it at the same time counts as an AEO in AEO_DEF_. It follows that the class of arguments selected by AEO_MF_ is the shared part of both frameworks. However, some AEOs that fit in AEO_DEF_ are simultaneously excluded from AEO_MF_.

Mizrahi’s thesis is about this set of arguments shared by both frameworks. His justification is sound and persuasive, as there is strong empirical evidence against the credibility of experts’ opinions, judgments, hunches, or intuitions (Mizrahi 2013, 2018)^8^. However, his thesis is misleading due to the idiosyncratic nature of his framework, and it is easy to regard it as referring to the entire AEO class, which is not the case. If the above reconstruction of Mizrahi’s framework is correct, then his thesis appears to be limited to a particular subset of the AEO class. After considering the framework-dependent differences, it occurs to be lacking in any controversy because it adjudicates the weakness of the argument class that is already regarded as weak by AEO_DEF_ standards. Put simply, Mizrahi claims that weak AEOs are weak arguments.

Moreover, Mizrahi implicitly agrees that arguments considered strong by AEO_DEF_ standards are indeed strong, but he arbitrarily excludes them from the AEO class. Mizrahi’s thesis adjusted to AEO_DEF_ is neither controversial nor undermines existing consensus on AEO strength. Therefore, whole debate on his claims is verbal.

## Footnotes

1 I decided to change Walton’s notion of “Major premise” to P1 as premise number one, and “Minor premise” to P2 as premise number two.

2 A list of critical questions matching the AEO scheme is quoted in the fourth section of this paper.

3 Liao’s accusations are conditional not simultaneous, which means that if Mizrahi argumentation is consistent, then it is irrelevant; if it is relevant, then it is inconsistent etc.

4 I assume that it is unknown to anyone, including expert *E*.

5 AEO are defeasible arguments, that means the probability of a conclusion being true, given the truth of premises cannot reach 1. New information can refute conclusion of any, even the strongest AEO.

6 Premises marked with “*” are fixed ones.

7 For example, arguments appealing to the opinion, unsupported by evidence and not shared by other experts in the field, of a personally unreliable and uncredible (e.g. biased) expert. However, it is open to interpretation if such arguments even are included in AEO_MF_ scheme.

8 Debate on Mizrahi’s papers unintentionally reveals an issue that demands to be addressed. The body of empirical evidence undoubtedly undermines the utility of Expertise Question in the AEO scheme. The question is to what extent?
